# Kynurenine pathway in kidney diseases

**DOI:** 10.1007/s43440-021-00329-w

**Published:** 2021-10-06

**Authors:** Izabela Zakrocka, Wojciech Załuska

**Affiliations:** Department of Nephrology, Medical University of Lublin, Jaczewskiego street 8, 20-954 Lublin, Poland

**Keywords:** Tryptophan, Kynurenine, Kynurenic acid, Chronic kidney disease, Acute kidney injury, Kidney transplant

## Abstract

Kidney diseases have become one of the most common health care problems. Due to a growing number of advanced aged patients with concomitant disorders the prevalence of these diseases will increase over the coming decades. Despite available laboratory tests, accurate and rapid diagnosis of renal dysfunction has yet to be realized, and prognosis is uncertain. Moreover, data on diagnostic and prognostic markers in kidney diseases are lacking. The kynurenine (KYN) pathway is one of the routes of tryptophan (Trp) degradation, with biologically active substances presenting ambiguous properties. The KYN pathway is known to be highly dependent on immunological system activity. As the kidneys are one of the main organs involved in the formation, degradation and excretion of Trp end products, pathologies involving the kidneys result in KYN pathway activity disturbances. This review aims to summarize changes in the KYN pathway observed in the most common kidney disease, chronic kidney disease (CKD), with a special focus on diabetic kidney disease, acute kidney injury (AKI), glomerulonephritis and kidney graft function monitoring. Additionally, the importance of KYN pathway activity in kidney cancer pathogenesis is discussed, as are available pharmacological agents affecting KYN pathway activity in the kidney. Despite limited clinical data, the KYN pathway appears to be a promising target in the diagnosis and prognosis of kidney diseases. Modulation of KYN pathway activity by pharmacological agents should be considered in the treatment of kidney diseases.

## Introduction

Kidney diseases represent various medical conditions, affecting more than 850 million people worldwide [1]. Among reported cases, chronic kidney disease (CKD) remains the most common kidney disorder, with poor outcome [2]. Overall, cardiovascular diseases are the predominant cause of death in patients with kidney function impairment [3], and there is an important need for effective prevention and introduction of early kidney damage biomarkers [4]. Oxidative stress, endothelial damage, and overactivity of the renin–angiotensin system (RAS) and adrenergic system are the main processes involved in the pathogenesis of kidney diseases [5, 6]. Although limited data are available, a role for the glutamatergic system in physiological and pathological conditions in kidneys has been reported [7].

**Fig. 1:**
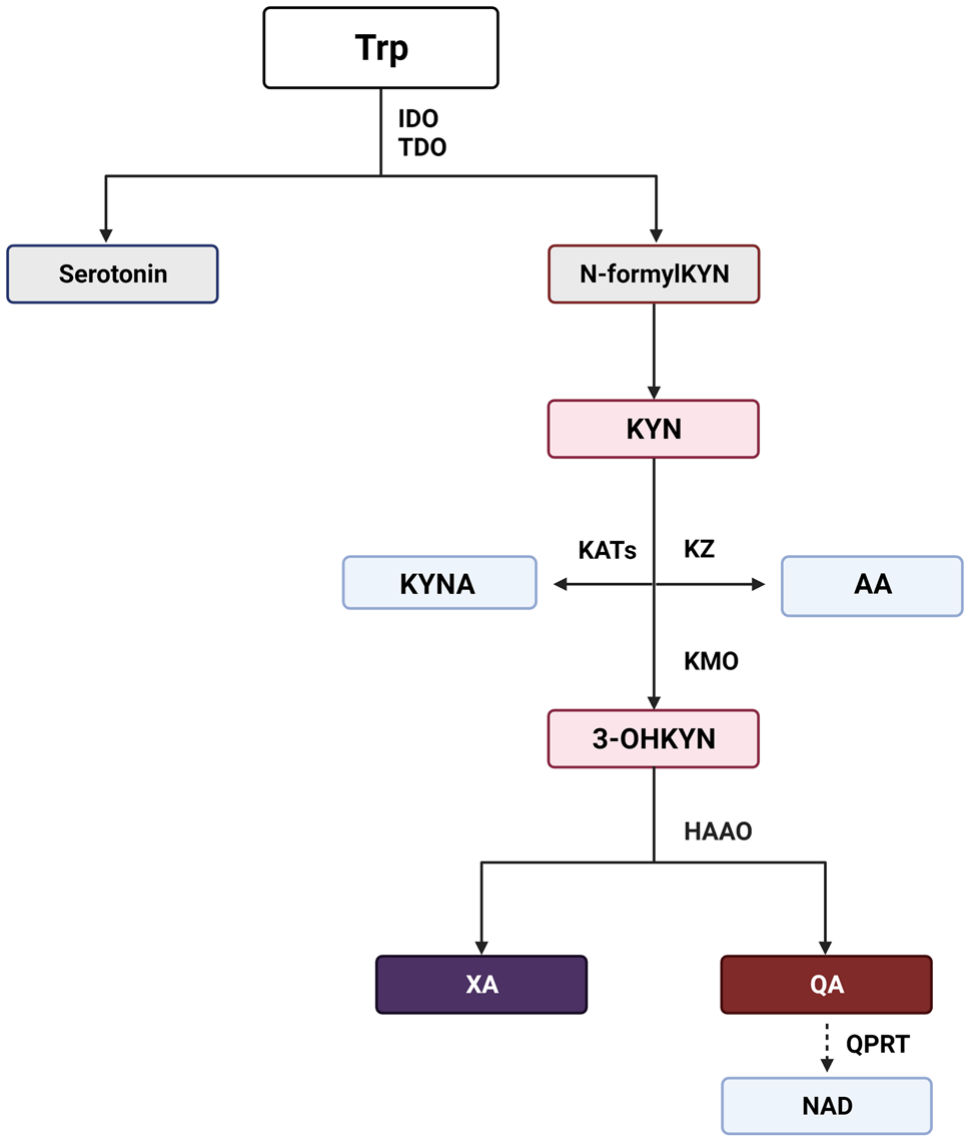
Kynurenine pathway. Tryptophan is degraded by IDO or TDO to serotonin or *N*-formylKYN. Later on, the formed KYN can be catabolized into 3-OHKYN, KYNA or AA. The 3-OHKYN is converted into XA and QA, with the final product NAD. *3-OHKYN* 3-hydroxykynurenine, *AA* anthranilic acid, *HAAO* 3-hydroxyanthranilic acid dioxygenase, *IDO* indoleamine 2,3-dioxygenase, *KAT* kynurenine aminotransferase, *KMO* kynurenine 3-monooxygenase (kynurenine 3-hydroxylase), *KYN* kynurenine, *KYNA* kynurenic acid, *KZ* kynureninase, *NAD* nicotinamide adenine dinucleotide, *N-formylKYN* *N*-formylkynurenine, *QA* quinolinic acid, *QPRT* quinolinic acid phosphoribosyltransferase, *TDO* tryptophan 2,3-dioxygenase, *Trp* tryptophan, *XA* xanthurenic acid. Created with BioRender.com

The kynurenine (KYN) pathway is the major route of tryptophan (Trp) metabolism, leading to the formation of many biologically active agents (Fig. 1) [8]. Conversion of Trp by indoleamine 2,3-dioxygenase (IDO), which is highly dependent on immune status, and tryptophan 2,3-dioxygenase (TDO), which is mainly expressed in the liver, is a key step in the KYN pathway [9]. TDO can be activated by higher substrate availability, glucocorticoids, reduced forms of nicotinamide adenine dinucleotide phosphate and kynurenic acid (KYNA) [10]. IDO can be found in most tissues, and under normal conditions, its activity is low; however, its activity can be enhanced by proinflammatory agents, such as tumour necrosis factor alpha (TNF-α), interleukin (IL)-6 and interferon gamma (IFN-γ) [11].

In ensuing steps, KYN is preferentially metabolized to 3-hydroxykynurenine (3-OHKYN) and then to 3-hydroxyanthranilic acid (3-OHAA), quinolinic acid (QA), and ultimately nicotinamide adenine dinucleotide (NAD) [12]. KYNA is produced by kynurenine aminotransferases (KATs) from the remaining KYN. Contrary to most Trp metabolites, which possess toxic properties, KYNA was shown to be neuroprotective, especially due to inhibition of *N*-methyl-d-aspartate and α-amino-3-hydroxy-5-methyl-4-isoxazolepropionate acid glutamate receptors, which are also widely distributed in the kidney [7]. Moreover, KYNA is a negative allosteric modulator of the alpha 7-nicotinic receptor and an agonist of G-protein-coupled receptors as well as aryl hydrocarbon receptors (AhRs) [12]. The role of KYNA in the regulation of renal haemodynamics [13] and heart rate [14] in spontaneously hypertensive rats was recently suggested. Owing to glutamatergic and cholinergic neurotransmission modulation, the KYN pathway has been broadly explored within the context of psychiatric and neurologic disorders [9]. However, the role of Trp metabolites in peripheral tissues, especially in the kidneys, is less well understood. KYN pathway metabolite accumulation has been shown in many reports concerning acute kidney injury (AKI) or CKD [10], and appropriate and clear conclusions indicating whether KYN metabolites have a direct effect on kidney damage are difficult to draw, because kidney filtration decreases in most nephrological disorders. Moreover, data establishing a role for the KYN pathway in physiological conditions are lacking. In this review, we aimed to analyse changes in KYN pathway activity reported in animal models of kidney diseases and in humans. Additionally, potential implications for kidney disease treatment are highlighted based on recently published studies.

## Kidney diseases

Here, we thoroughly review the relationship between KYN pathway activity and the most common kidney disorder, CKD, with special attention given to diabetic kidney disease, AKI, glomerulonephritis and monitoring kidney graft function. Due to the increasing number of renal cancer patients, the potential involvement of the KYN pathway in this condition is discussed. Finally, current knowledge about drugs affecting KYN pathway activity is presented.

### Chronic kidney disease (CKD)

CKD is one of the most common health care problems, affecting more than 10% of the general population [15]. The incidence of CKD is increasing due to population ageing and higher rate of concomitant disorders. Diabetes mellitus is the most common cause of CKD, leading to kidney replacement therapies in half of these patients [16]. The mortality of patients with CKD receiving dialysis is comparable to that of patients with some solid organ cancers [17] and is predicted to increase in the coming years [18]. Among the mechanisms involved in CKD pathogenesis, chronic inflammation and autonomic dysfunction are of special importance [15]. Overall, decreased excretion of waste products and impaired metabolic kidney function contribute to inflammatory conditions during the CKD course [19]. Therefore, the KYN pathway seems to play a crucial role in CKD. Although several reports have been published on this topic [20, 21], the articles published by Pawlak et al. has greatly broadened our knowledge about KYN pathway metabolite changes in CKD [10].

**Fig. 2:**
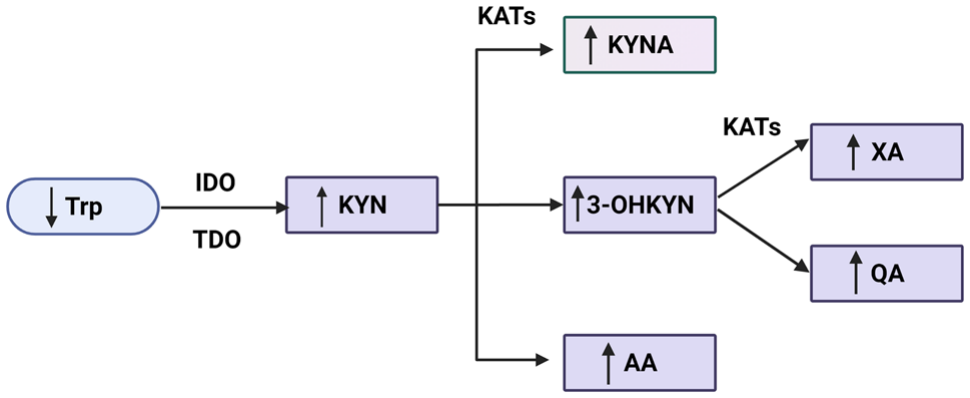
Changes in kynurenine pathway during chronic kidney disease. In chronic kidney disease was found lower serum concentration of Trp in both, animal and human studies. Conversely, higher serum KYN, KYNA, 3-OHKYN, AA, XA and QA were reported, suggesting kynurenine pathway activation in chronic kidney disease. 3-*OHKYN* 3-hydroxykynurenine, *AA* anthranilic acid, *IDO* indoleamine 2,3-dioxygenase, *KAT* kynurenine aminotransferase, *KYN* kynurenine, *KYNA* kynurenic acid, *QA* quinolinic acid, *TDO* tryptophan 2,3-dioxygenase, *Trp* tryptophan, *XA* xanthurenic acid. Created with BioRender.com

In the experimental model of CKD in nephrectomized rats, lower plasma Trp levels were detected, whereas plasma 3-OHKYN, KYNA, xanthurenic acid (XA), anthranilic acid (AA) and QA concentrations were elevated (Fig. 2) [22]. Interestingly, accumulation of Trp metabolites was shown to be proportional to kidney function decline [23]. Moreover, kidney KAT and kynurenine 3-monooxygenase (KMO) activity in the rat kidney was increased, but the activities of kynureninase (KZ) and 3-hydroxyanthranilic acid dioxygenase (HAAO) were significantly lower in advanced renal failure [23]. In another study, elevated renal KMO activity was confirmed [24] and responsible for high KYN and 3-OHKYN levels in a kidney failure model.

In patients with CKD, plasma Trp was found to be decreased by 40–60%; KYN and 3-OHKYN increased by 32–96% and 184–306%, respectively [25]. The plasma 3-OHKYN/KYN ratio was reported to be higher by approximately 40–154% in uraemic patients, whereas the KYN/Trp ratio significantly increased by 140–240%, indicating IDO activation [25]. Moreover, a significant elevation of Cu/Zn superoxide dismutase activity, total peroxide and high sensitivity C-reactive protein levels were observed, and KYN with 3-OHKYN correlated positively with these oxidative markers in CKD patients. Similarly, Schefold et al. reported higher IDO activity in CKD patients in association with disease severity and levels of inflammatory markers, such as C-reactive protein or soluble TNF-receptor-1 [26], as summarized in Fig. 2. Administration of lipid-lowering drugs, which have anti-inflammatory properties, significantly decreased plasma KYN and the KYN/Trp ratio in CKD patients [27, 28].

In addition to observed disturbances in KYN pathway activity during CKD, the link between CKD complications and Trp metabolites has been presented in multiple studies (Table 1). Levels of KYN metabolites are related to neurological symptoms, impaired erythropoiesis and erythrocyte structure, atherosclerosis, thrombosis, hyperfibrinolysis and bone damage during CKD.

**Table 1:** Complications of chronic kidney disease related with kynurenine pathway activity

Complication	Kynurenine pathway activity impairment	References
Cognitive impairment	High plasma KYN in patients with CKD	[123]
Anxiety, depression	High plasma indole-3-acetic acid in patients with CKD	[123]
Anaemia	3-OHKYN during hypoxia inhibits erythropoietin release from liver hepatocellular carcinoma cells	[124]
	Plasma 3-OHKYN level in CKD patients negatively correlated with haematocrit, haemoglobin and red blood cells count	[124]
	AA related with erythrocytes damage by decreasing their osmotic resistance	[125]
Atherosclerosis	Plasma KYN, QA and QA/KYN ratio in patients with CKD associated with intima-media thickness (IMT)	[126, 127]
	Higher mean carotid artery IMT, whereas ankle-brachial pressure index lower in high plasma KYN/Trp hemodialyzed patients	[128]
	High KYNA and KYNA/KYN ratio positively related with hyperhomocysteinemia and IMT in continuous ambulatory peritoneal dialysis patients	[129]
Thrombosis	High plasma thrombomodulin and von Willebrand factor positively associated with plasma KYN, 3-OHKYN, QA as well as Cu/Zn superoxide dismutase activity and malondialdehyde level	[130]
	High plasma tissue factor level and TF/tissue factor inhibitor ratio in CKD patients positively related with 3-OHKYN, AA, KYN/Trp and 3-OHKYN/KYN ratios	[131, 132]
	High plasma AA level related with increased monocyte chemoattractant protein-1 concentration	[133]
	High plasma soluble intercellular adhesion molecule-1, soluble vascular adhesion molecule-1 related with KYN, KYNA and Cu/Zn superoxide dismutase	[134]
Hyperfibrinolysis	High soluble urokinase plasminogen activator receptors activity associated with high plasma KYN	[135]
Bone damage	High plasma KYN negatively related with the main parameters of bone biomechanics, bone geometry, and bone mineral density values in subtotal 5/6 nephrectomized rats	[31]
Muscle damage	High KYN and KYNA level negatively impact on mitochondrial energy transfer, due to complex III and IV impairment	[136]

A novel concept linking the KYN pathway and diseases related to CKD has also been discussed. Trp metabolites, especially KYNA, are known endogenous AhR ligands [29]. Given that AhR activation mediates cardiotoxicity and vascular damage through procoagulant and prooxidant phenotypes [30], the effects of KYN metabolite accumulation on the cardiovascular system in CKD patients can in part be explained by AhR activity modulation. A link between AhR activation and bone damage in CKD was shown in an animal model [31]. Interestingly, a potential role of the gut microbiota in Trp degradation, AhR activation and kidney fibrosis was recently discussed [32]. Disturbance in the gut flora may also have a bidirectional effect on kidney function by attenuating inflammatory conditions on the one hand and leading to insulin resistance, immune disorders, and atherosclerosis on the other hand [32].

Finally, the role of a specific type of kidney replacement therapy in lowering serum KYN levels and their metabolites has been analysed. Although both haemodialysis and peritoneal dialysis were shown to decrease levels of Trp metabolites, their effectiveness appears to be different [10]. This indicates that both kinds of dialysis do not fully restore abnormalities of the KYN pathway in CKD patients and do not equally protect them from possible CKD complications. Importantly, Yilmaz et al. reported higher IDO activity and oxidative stress in patients undergoing peritoneal dialysis compared to haemodialysis, which may have detrimental effects on CKD course [33].

#### Diabetic nephropathy

Diabetes mellitus is the predominant cause of CKD worldwide [6]. Indeed, approximately 30–40% of patients with diabetes will develop diabetic kidney disease. Albuminuria remains an initial screening test for diabetic nephropathy [34]. Proteomic analysis, including KYN pathway metabolites, is an interesting and promising diagnostic tool in this kind of kidney damage. The first findings concerning abnormalities in the KYN pathway in streptozocin-induced diabetic rats were reported by Smith and Pogson [35], who observed increased activity of TDO and KZ in isolated liver cells of diabetic animals of 2.5-fold and 3.3-fold, respectively, suggesting higher degradation of Trp to KYN metabolites. Another study reported that XA, which binds to insulin, and QA, which decreases proinsulin secretion and suppresses insulin release play a crucial role in the pathogenesis of diabetes [36]. Interestingly, IDO expression and its enzymatic activity were found to be upregulated in human isolated islets by IFN-γ, leading to increased KYN production [37]. These effects were inhibited by IL-4 and 1-alpha-methyl-Trp, an IDO inhibitor [37]. However, the authors focused on the time of islet IFN-γ exposure. Short-term IFN-γ effects may prevent cytotoxic damage, but longer IFN-γ exposure and overproduction of Trp metabolites may be responsible for cell destruction. A similar opinion was presented by Oxen rug, suggesting that chronic inflammation, involved in the pathogenesis of all components of metabolic syndrome, including diabetes mellitus, leads to ‘superinduction’ of IDO, as well as TDO, and overactivation of the KYN pathway [38].

In diabetic patients, higher levels of KYN, 3-OHKYN, and xanthurenic acid (XA) were detected in both plasma and urine [36]. However, the results of studies on diabetic nephropathy differ due to the diversity of the analysed groups. First, the presented data differed among the types of diabetic patients examined. Clinical analysis of serum markers in patients with type 1 and type 2 diabetes revealed important differences between these groups of patients [39]. A significant increase in Trp and AA levels was observed in diabetes type 1 patients, though KYN concentrations were similar between the examined groups. The KYNA serum level was higher in both diabetic groups. The authors have suggested that downregulation of IDO by AA occurs in diabetes type 1, leading to Trp elevation and autoimmune process dysregulation. Second, different results can be observed in patients with various levels of albuminuria and kidney function decline. For example, in an analysis of 30 patients with normoalbuminuria and 55 (33 with normal glomerular filtration rate [GFR] and 22 with reduced GFR) with new-onset microalbuminuria due to diabetes mellitus type 1, serum *N*-formyl KYN levels were lower in microalbuminuria patients, but without kidney function decline [40]. In contrast, Hirayama et al. performed capillary electrophoresis coupled with time-of-flight mass spectrometry to search for serum markers of diabetic nephropathy with a high level of albuminuria (urine albumin-to-creatinine ratio > 300 mg/g) [41]. Among 289 metabolites analysed, they identified 19 substances, including creatinine, aspartic acid, γ-butyrobetaine, citrulline, symmetric dimethylarginine, KYN, azelaic acid, and galactaric acid that could distinguish patients with and without albuminuria. The serum KYN level was significantly elevated in patients with diabetic nephropathy and correlated positively with the urine albumin-to-creatinine ratio and negatively with GFR. It should be noted that the group of patients with high albuminuria levels also had greater kidney function decline, significantly affecting the results presented. Additionally, serum IDO activity was significantly higher in diabetes type 2 patients and closely related to GFR (especially when GFR > 60 ml/min/1.73 m^2^), but with no correlation with patient age [42]. Moreover, IDO activity seemed to be more elevated in diabetes than in patients with glomerulonephritis; however, it was much lower than in patients undergoing haemodialysis, pointing to very high immunization of this group of patients. Additionally, Oxenkrug showed elevated serum levels of KYN, KYNA and XA in diabetic patients, indicating their possible use as diabetic biomarkers [43]. In patients with CKD caused by diabetes mellitus, plasma Trp levels are inversely related to CKD stages, whereas KYN, KYNA, and QA correlate positively with disease severity and lower kidney filtration [44]. Neither TNF-α nor IL-6 are related to the KYN/Trp ratio, but TNF-α is associated with the KYN level, indicating inflammatory system activation in diabetic nephropathy. Although Trp and the KYN/Trp ratio were shown to be associated with albuminuria, only the KYN/Trp ratio has been proven to predict response to drugs lowering albuminuria levels in diabetic kidney disease [45].

In addition to serum testing, interesting results were reported in an analysis of urine in diabetes type 2 patients [46]. A protein profile of morning urine samples tested by liquid chromatography–mass spectrometry revealed a decrease in urine Trp and KYNA concentrations and higher KYN levels in diabetic patients, probably in part due to different GFRs and lower filtration of these metabolites. Furthermore, high urinary XA excretion has been found in patients with prediabetes [47] and diabetes type 2 [48]. High urine KYN, KYNA, XA, and QA concentrations and a high KYN/Trp ratio are also present in patients with metabolic syndrome, especially those under 60 years of age [49].

#### Acute kidney injury (AKI)

AKI is a very common clinical problem, especially in hospitalized patients, affecting 57.3% of patients in intensive care units [50]. The mortality of AKI is estimated to be 26.9% and increases up to 55.3% among patients who need kidney replacement therapy [51]. In general, prevention and treatment of AKI highly depend on the factors responsible for kidney damage [52]. Due to nonspecific symptoms and several clinical parameters defining AKI, appropriate diagnosis can be delayed, which may lead to irreversible kidney damage and CKD. The standard laboratory marker used to monitor renal function, i.e. serum creatinine, is useless in elderly or malnourished patients as well as in rapid decline in kidney function [50]. Due to inconclusive results of studies in AKI animal models and the nonavailability of most potential AKI biomarkers in clinical practice [53, 54], the search for other AKI indicators is crucial. Although involvement of the KYN pathway in AKI has been analysed, most data are from animal studies, and different animal models of AKI may lead to various conclusions.

Immune-mediated and toxic AKI remain the most common AKI models. Saito et al. showed that gerbils subjected to pokeweed mitogen-induced multiorgan damage manifest an increase in renal IDO activity, serum KYN concentration and higher serum and urinary QA levels, whereas the activity of other enzymes in the kidney, such as KMO, KZ, KAT and HAAO, did not significantly change [55]. On the other hand, Zheng et al. showed better kidney function and reduced tubular damage and macrophage infiltration in KMO^null^ mice after renal ischaemia reperfusion injury [56]. In cisplatin-induced AKI in C57BL/6 mice, galectin 3 expressed on renal dendritic cells was shown to protect against kidney damage by promoting Toll-like receptor-2-dependent activation of IDO and the KYN pathway in renal dendritic cells [25]. Moreover, higher expansion of Tregs in injured kidneys was found, pointing to an interesting mechanism of cisplatin-induced AKI as well as other potential methods of immune-mediated kidney injury prevention.

Zgoda-Pols et al. showed that KYN and KYNA serum concentrations were significantly elevated in a toxic AKI model of mice with kidney damage caused by the nicotinic agonist SCH 900424 [57]. Similarly, elevated serum KYN levels were reported in glycerol-induced AKI in rats, which probably led to KYNA elevation, as activity of multidrug resistance-associated proteins, known to be blocked by KYNA, was inhibited [58]. In gentamicin-induced AKI in rats, a higher urinary Trp level was found, whereas the KYNA concentration in the urine decreased, which may be in part related to impaired kidney function and a lower KYNA filtration rate [59, 60]. KYNA has also been identified as a biomarker of AKI caused by arystocholic acid [61, 62] and mercuric chloride [63]. Despite limited data, KYNA can be considered a nephroprotective agent. In ischaemia–reperfusion AKI in rats, KYNA, together with ketamine and magnesium sulphate, was shown to attenuate kidney impairment and oxidative stress [64]. KYNA was also shown to prevent AKI in rats exposed to heatstroke [65].

Contrary to animal studies, data on the significance of the KYN pathway in AKI in humans are limited due to ethical considerations. Interesting results concerning the KYN pathway in a contrast nephropathy were published by Reichetzeder et al. [66]. In a prospective cohort study, 245 patients were followed for 120 days after contrast media administration during coronary angiography. Although preinterventional serum KYN levels were not related to a higher risk of AKI, KYN concentration was significantly associated with kidney function impairment in a long-term observation [37]. On the other hand, among critically ill patients with AKI, urinary Trp, KYN, AA, serotonin concentration and KYN/Trp ratio were significantly lower in a group with late-/nonrecovery kidney function, though a high KYNA urinary level was observed [67]. Moreover, KYNA was associated with a higher AKI stage, longer AKI duration, the need for renal replacement therapy and 30-day mortality. Similarly, Dąbrowski et al. observed high plasma KYNA levels in septic shock patients with AKI to be tightly associated with outcomes and predicted higher mortality [68].

#### Glomerulonephritis

Glomerulonephritis is one of the most common causes of CKD. Complement activation and chronic inflammation leading to tissue fibrosis are crucial mechanisms of glomerular injury [69]. In many cases, the cause of glomerular damage cannot be established, and the pathogenic process is irreversible, despite the use of broad-spectrum immunosuppressive drugs. New glomerular targets may help improve diagnostic and therapeutic approaches, in conjunction with kidney biopsy and available serological markers, such as phospholipase A_2_ receptor antibodies in membranous nephropathy [70]. Elevation of serum KYN levels in patients with glomerulonephritis and normal kidney function has been reported [71]; a high neopterin concentration was simultaneously observed, suggesting immunological system involvement in KYN pathway activation. In cultured mesangial cells, Yoshimura et al. showed that AA and 3-OHKYN suppresses proliferation but QA and KYN promote proliferation, though not at higher concentrations [72]. In an animal model of immunoglobulin A (IgA) nephropathy, Yang et al. found that intraperitoneally administered IDO inhibitor 1-methyl-Trp increased renal damage and IgA accumulation in glomeruli and upregulated Th/Treg expression and cytokine release [73]. In contrast, the IDO agonist ISS-ODN significantly decreased renal tissue damage and IgA accumulation as well as Th17-mediated cytokine changes in an IgA nephropathy model. In humans with biopsy-proven IgA nephropathy, urinary AA correlates significantly with proteinuria, rendering it a potential noninvasive biomarker of proteinuria severity [74].

The involvement of KYN in mesangioproliferative glomerulonephritis has also been discussed [75, 76]. In an animal model of crescentic glomerulonephritis and nephrotoxic serum nephritis, Hou et al. observed an increased IDO activity (by the KYN/Trp ratio) in serum and renal tissue and upregulated IDO gene expression in glomeruli and tubular epithelial cells [77]. Moreover, 1-methyl-Trp increased glomerular crescent formation, macrophage infiltration, and intrarenal cell proliferation, significantly stimulating nephritis progression. Similarly, high serum KYN levels with decreased Trp concentrations were found in patients with antineutrophil cytoplasmic antibody-associated vasculitis, one of the causes of rapid progressive glomerulonephritis with crescent formation [78]. In an autoimmune glomerulonephritis model in Wistar–Kyoto rats, a model of anti-glomerular basement membrane glomerulonephritis, 3-OHAA and 3-OH KYNA were shown to decrease glomerular damage and inflammatory cell infiltration, decrease proteinuria and improve kidney function [79]. These studies provide significant evidence of KYN pathway involvement in glomerulonephritis pathogenesis; however, many data are lacking, especially concerning immune-mediated membranous nephropathy [80] or focal segmental glomerulosclerosis [81, 82], which are disorders with severe clinical manifestations, variable sensitivity to immunosuppression and uncertain prognosis.

#### Kidney transplantation

Kidney transplantation remains the best form of kidney replacement therapy for CKD. A substantial increase in quality of life and survival after kidney transplantation in comparison with haemodialysis patients has been repeatedly proven [83]. Nevertheless, it should be noted that an increased risk of opportunistic infections [84] and cancers [85] due to immunosuppressive therapy may limit benefits in some patients. Another crucial problem is kidney transplant rejection because of insufficient immunosuppression, recurrent opportunistic infections and reoccurrence of primary kidney disease [86]. This highlights the need to search for novel serological, urine-derived and histological markers to predict outcomes after kidney transplantation [87]. Because KYN pathway activity is regulated by the immunological system [88], it is an interesting target to monitor kidney graft function. For instance, Myśliwiec et al. observed higher concentrations of plasma KYN metabolite levels in kidney transplant recipients, albeit lower than in patients on haemodialysis [89]. Despite a negative correlation between Trp and creatinine concentration, a positive correlation was found between Trp and GFR in these patients, showing higher Trp degradation when kidney function deteriorates [89]. Furthermore, this study showed that kidney transplantation significantly decreased KYN levels in comparison with CKD patients. Similar findings were reported by Lahdou et al., who observed lowered plasma KYN concentrations in patients after kidney transplantation [90]. Moreover, it was shown that an elevation in KYN levels after transplantation predicts acute renal graft rejection but that elevation before kidney implantation does not have any role in kidney function prediction. Similarly, Holmes et al. reported significantly elevated levels of serum KYN in patients with acute graft rejection or during viral or bacterial infection [91]. Although the KYN concentration appears not be related to the creatinine concentration and is not affected by corticosteroid therapy, in a retrospective analysis, KYN was proven to be an effective marker of acute kidney rejection in conjunction with serum creatinine levels. Because IDO activation by IFN-γ depletes Trp and suppresses T-cell mediated immunity, the possible role of IDO in kidney graft control was analysed by Brandacher et al. [92]. An elevated KYN/Trp ratio was observed in kidney transplant patients, though the serum and urine KYN/Trp ratio was greatly increased in graft rejection. Importantly, higher IDO immunostaining was detected in rejected kidney biopsies, especially in tubular epithelial cells. Based on 2-year follow-up, Vavrincova-Yaghi et al. observed that both serum and urine KYN/Trp ratios can be useful for long-term graft function monitoring [93]. In kidney biopsies performed after 2 years in patients with previous acute, borderline or chronic allograft rejection, IDO was mainly found in inflammatory cells and glomeruli, correlating with serum and urinary findings. In another study performed by de Vries et al., serum 3-OHKYN was the strongest predictor of graft failure and patient mortality [94], and Minović et al. reported increased risk of death due to cancer or infections in addition to the relationship between 3-OHKYN and kidney graft recipient all-cause mortality [95].

The correlation between viral infections after kidney transplantation and the KYN pathway was analysed in a study by Sadeghi et al. [96]. Contrary to polyomavirus BK, cytomegalovirus infection was strongly associated with higher plasma KYN and QA concentrations as well as disease severity. To help differentiate between acute graft rejection and severe infection, Dharnidharka et al. performed a study on 29 children who received kidney transplant in 1 year of observation [97]; although the serum KYN/Trp ratio was significantly higher in those who experienced acute graft rejection, peripheral blood CD4-ATP levels were useful in differential diagnosis, because they were lower in the group with infections. Additionally, Kaden et al. showed higher plasma KYN levels in kidney graft recipients with cytomegalovirus infection and its relationship with disease severity [98].

Contrary to previous findings, there are some data disputing the connection between the KYN pathway and graft rejection. In a retrospective study involving 307 kidney graft recipients, no association between KYN concentration and primary renal disease, graft rejection or survival was observed [99]. Regardless, pretransplant KYN levels were higher in panel-reactive antibody-positive patients and those waiting longer on a transplant list. On the other hand, a lower pretransplant KYN concentration was related to faster graft function occurrence. Although the results of this study point to the link between the KYN pathway and the immunization status of patients before kidney transplantation, the study was conducted with the use of a photometric method to quantify KYN levels and not high-sensitivity high-performance liquid chromatography or liquid chromatography–mass spectrometry, as in most studies, which may be an important limitation of this report.

### Kidney cancer

To date, the role of Trp metabolites in carcinogenesis has not been unequivocally established. There are only a few studies linking the KYN pathway with kidney cancer. Teulings et al. reported a high urine concentration of 3-OHAA in patients with untreated kidney cancer [100]. Similarly, elevated urinary levels of 3-oxyAA were found in patients with renal cell carcinoma (RCC), though changes in 3-oxyKYN were statistically insignificant [101]. Contrary to previous studies, Sato et al. postulated that elevated urinary KYN concentration is a predictive marker of RCC malignancy [102]. In another study, elevated serum KYN and 3-OHAA concentrations were found in a group of 24 patients treated with IFN-γ for metastatic carcinoma, with a concomitant decrease in KYNA levels [103]. Moreover, Trott et al. reported an interesting correlation between IDO activity and carcinogenesis [104]. In addition to stimulation of Trp degradation in RCC, they showed increased expression of IDO not only in human cancer cells, but also in the RCC environment. KYN metabolites and IDO inhibitors alone did not affect RCC cell or murine renal cell adenocarcinoma cell survival or proliferation in vitro. However, IFN-α together with methyl-thiohydantoin-dl-Trp, an IDO inhibitor, decreased renal cancer cell growth, highlighting the role of IDO inhibitors as potential anticancer agents. In the analysis of 40 human clear cell RCC samples, higher IDO expression, AhR expression, KYN levels and KYN/Trp ratios were found [105]. Moreover, serum KYN was significantly elevated in RCC patients, whereas the KYN/Trp ratio was linked to clinical stage, tumour size, Fuhrman grade, lymph node involvement and visceral metastases. More evidence about enzymatic dysregulation of the KYN pathway in RCC was provided by Hornigold et al. [106]. In contrast to IDO upregulation, a decrease in quinolinic acid phosphoribosyltransferase (QPRT) and HAAO expression was detected. Although expression of most final enzymes in the KYN pathway was decreased, that of nicotinamide phosphoribosyltransferase, a crucial enzyme in the NAD salvage pathway, was increased in RCC. This interesting observation again shows dysregulation of the KYN pathway in renal cancer cells and highlights possible targets in anticancer treatment.

As AhR overexpression in RCC cells has been reported, KYNA, an AhR agonist, seems to be involved in carcinogenesis regulation. A lower KYNA content in RCC tissue was found [107], which in part can be related to loss of filtration by a cancer tissue. On the other hand, it was shown that expression of human organic anion transporters 1 and 3, responsible for KYNA uptake, is markedly decreased in RCC tissue [108]. Importantly, Walczak et al. demonstrated antiproliferative and antimigrative effects of KYNA on RCC Caki-2 cells [107]. KYNA is also reported to inhibit cancer cell signalling, including p38 mitogen-activated protein kinase involved in cell cycle regulation [107]. Interestingly, KYNA may inhibit multidrug resistance-associated protein 4 and breast cancer resistance protein, transporters responsible for drug resistance [109, 110], possibly enhancing responsiveness to anticancer drugs, as presented by Walczak et al. [111].

### Drugs affecting kynurenine pathway in the kidney

**Fig. 3:**
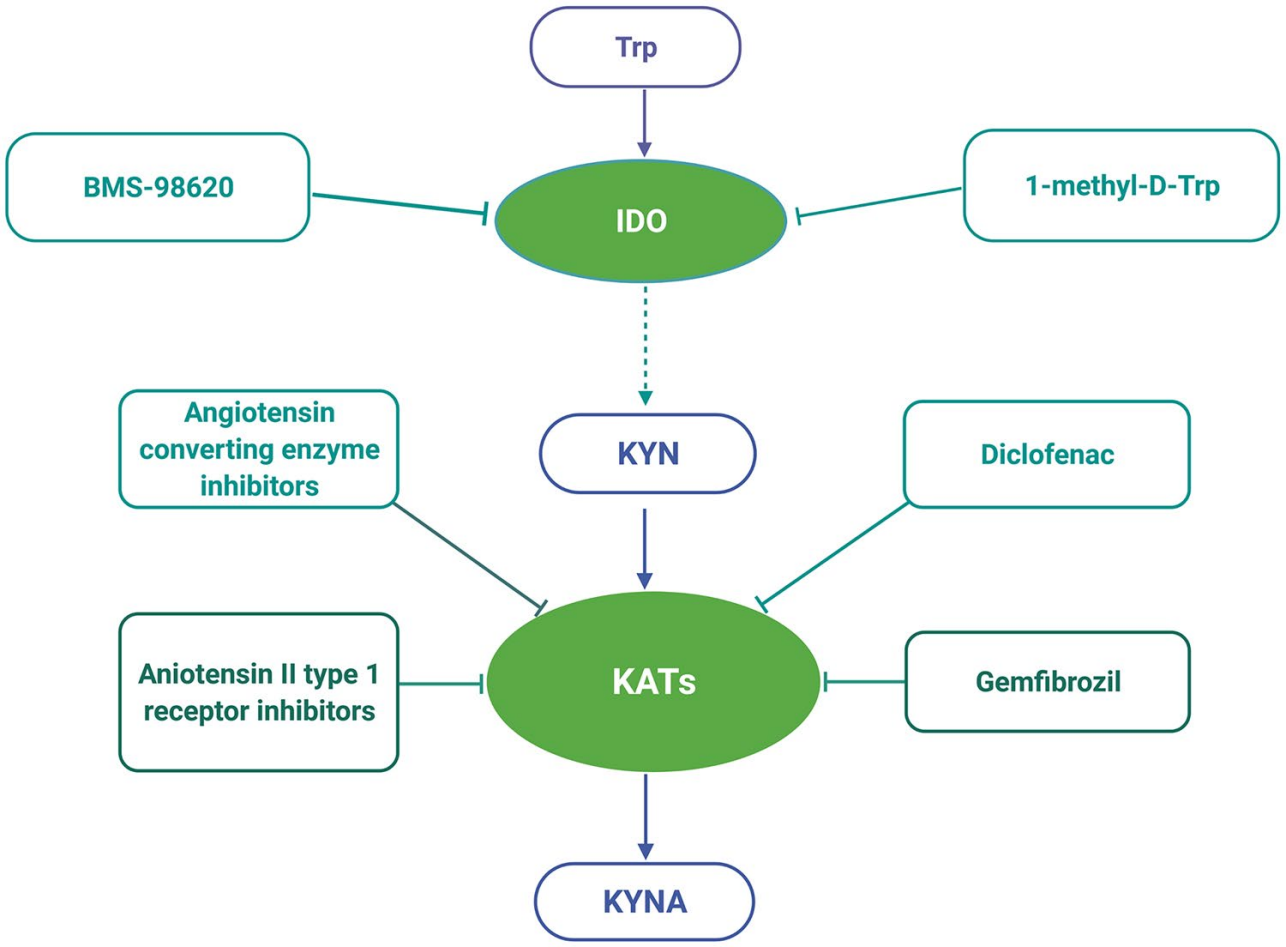
Drugs affecting kynurenine pathway in the kidney. Two IDO inhibitors—BMS-98620 and 1-methyl-d-Trp, as well as KAT inhibitors—angiotensin converting enzyme inhibitors, angiotensin II type 1 receptor antagonists, diclofenac and gemfibrozil, are recently described modulators of kynurenine pathway in the kidney. *IDO* indoleamine 2,3-dioxygenase, *KAT* kynurenine aminotransferase, *KYN* kynurenine, *KYNA* kynurenic acid, *Trp* tryptophan. Created with BioRender.com

Based on the available literature, the KYN pathway seems to be an important regulator of kidney function under normal conditions, as well as in renal diseases, and drugs influencing KYN degradation may be promising agents in kidney disorder treatment. Nonetheless, data about substances affecting KYN metabolism in the kidney are very limited (Fig. 3).

As mentioned previously, 1-methyl-d-Trp, an IDO inhibitor, causes renal damage in animal models of IgA nephropathy [73] and mesangioproliferative glomerulonephritis, probably due to immune dysregulation [77]. In contrast, IDO inhibition and, as a result, a reduction in KYN formation were reported to prevent ischaemia–reperfusion injury kidney damage in mice [112]. Unfortunately, these findings were discredited by Čepcová et al., who presented an IDO-independent nephroprotective effect of 1-methyl-d-Trp in an ischaemia reperfusion model [113]. The beneficial mechanisms of 1-methyl-d-Trp may include decreased toll-like receptor 4 signalling, impaired transforming growth factor beta signalling and epithelial–mesenchymal transition, and inappropriate activation of tubular epithelial cells, dendritic cells, NK cells and T cells. Interestingly, in a recently published study, the antifibrotic effect of 1-methyl-d-Trp and another IDO inhibitor, BMS-98620, was confirmed in murine kidney slices and in mice with unilateral ureteral obstruction [114]. Based on these reports, we may conclude that pharmacological modulation of IDO activity remains an interesting approach for preventing kidney damage, especially under conditions related to tissue fibrosis, such as CKD.

Another potentially interesting group of agents in kidney function regulation are KAT inhibitors. Edwards and Mather reported that diclofenac given subcutaneously significantly increased kidney KYN and KYNA levels in rats [115]. KYN and KYNA accumulation was suggested to be caused by inhibition of renal elimination. However, another mechanism of action was reported by Zakrocka et al. [116]. In rat kidney homogenates, in vitro diclofenac was found to inhibit KAT activity and KYNA production, similar to gemfibrozil in another study [117]. Similarly, angiotensin converting enzyme inhibitors and angiotensin II type 1 receptor blockers (ARBs) were observed to modify kidney KYNA levels. ACE-I lowers the KYNA concentration in rat kidney homogenates in vitro without affecting KAT activity [118], and ARBs decrease KYNA production by KAT inhibition in rat kidneys in vitro [119, 120]. Unfortunately, although Chmiel-Perzyńska et al. showed that after 4 weeks of administration, losartan decreases brain KYNA concentration in a rat model of diabetes mellitus, data on the long-term effect of ARBs on kidney KYN pathway activity are lacking [121]. In particular, the effect of ARBs on KYNA synthesis seems to be of great importance and can be in part related to their mechanism of nephroprotection. Interestingly, Cernaro et al. observed lower serum KYN levels in patients with diabetic nephropathy taking angiotensin converting enzyme inhibitors or ARBs [122]. Additionally, the relationship between KYN and albuminuria, proteinuria and GFR diminished in patients taking these drugs, suggesting their beneficial role in kidney function preservation. The plasma KYN/Trp ratio is also reported to be significantly associated with albuminuria and ARB responsiveness in diabetic kidney disease patients [45].

The presented results indicate a great need for further studies analysing agents that potentially affect the KYN pathway and can be used in both diagnostic and therapeutic procedures.

## Conclusions

The KYN pathway is a promising target in kidney disease prevention and treatment. Although many questions remain to be answered, future studies should explicitly explain the role of the KYN pathway in the pathogenesis of renal disorders, especially CKD. Searching for novel agents modulating KYN pathway activity may contribute to the introduction of new drugs for kidney diseases and significantly improve patient prognosis.

## Abbreviations

3-OHAA: 3-Hydroxyanthranilic acid
3-OHKYN: 3-Hydroxykynurenine
AA: Anthranilic acid
AhR: Aryl hydrocarbon receptor
AKI: Acute kidney injury
ARB: Angiotensin II type 1 receptor blocker
CKD: Chronic kidney disease
GFR: Glomerular filtration rate
HAAO: 3-Hydroxyanthranilic acid dioxygenase
IDO: Indoleamine 2,3-dioxygenase
IFN-γ: Interferon gamma
IgA: Immunoglobulin A
IL: Interleukin
IMT: Intima-media thickness
KAT: Kynurenine aminotransferase
KMO: Kynurenine 3-monooxygenase
KYN: Kynurenine
KYNA: Kynurenic acid
KZ: Kynureninase
NAD: Nicotinamide adenine dinucleotide
QA: Quinolinic acid
QPRT: Quinolinic acid phosphoribosyltransferase
RAS: Renin–angiotensin system
RCC: Renal cell carcinoma
TDO: Tryptophan 2,3-dioxygenase
TNF-α: Tumour necrosis factor alfa
Trp: Tryptophan
XA: Xanthurenic acid
